# Permanent Draft Genome Sequence of the French Bean Symbiont *Rhizobium* sp. Strain RSm-3 Isolated from the Eastern Himalayan Region of India

**DOI:** 10.1128/genomeA.00175-17

**Published:** 2017-04-13

**Authors:** Ritu Rai, Erik Swanson, Indrani Sarkar, Dorjay Lama, Feseha Abebe-Aleke, Stephen Simpson, Krystalynne Morris, W. Kelley Thomas, Pallab Kar, Maher Gtari, Arnab Sen, Louis S. Tisa

**Affiliations:** a Department of Botany, Bioinformatics Facility, University of North Bengal, Siliguri, India; b University of New Hampshire, Durham, New Hampshire, USA; c St. Joseph College, Darjeeling, India; d Université de Tunis El Manar, Tunis, Tunisia

## Abstract

The genus *Rhizobium* contains many species able to form nitrogen-fixing nodules on plants of the legume family. Here, we report the 6.9-Mbp draft genome sequence of *Rhizobium* sp. strain RSm-3, with a G+C content of 61.4% and 6,511 candidate protein-coding genes.

## GENOME ANNOUNCEMENT

The genus *Rhizobium*, established in 1889, is a group of motile, aerobic, and Gram-negative bacteria in the alphaproteobacterial group with a moderate G+C percentage (60%) (1, 2). Members of the genus *Rhizobium* form a symbiotic association with various legume plants of the Fabaceae family (3–5) and form nodules on the root surface. These nodules are the sites of nitrogen fixation. The symbiosis between *Rhizobium* and legumes is of great importance (6). Compared to the use of chemical fertilizers, symbiosis offers cheaper and more effective agronomic practices by providing an adequate supply of N for legume-based crops (7, 8). The French bean, or common bean (*Phaseolus vulgaris* L.), is one of the most important plant hosts of *Rhizobium* spp., with the broadest genetic base (9, 10), and is one of the major cultivated crops containing large amounts of protein, minerals, and antioxidant compounds (11).

*Rhizobium* sp. strain RSm-3 was isolated from the root nodules of *P. vulgaris* collected from the Sonada region of Darjeeling district (26.9400°N, 88.250°E; altitude, 5,157 ft) of West Bengal, India. The strain showed antagonistic activity against the fungal pathogen *Fusarium solani* and resistance against most of the antibiotics tested against it. These interesting features led us to do 16S rRNA gene sequencing, which identified the strain as *Rhizobium* sp. and shared 99% identity with *Rhizobium etli* EBRI 21 (accession no. AY221176.1). This strain was sequenced to provide a greater understanding of these physiological properties and its interaction with *P. vulgaris*.

Sequencing of the draft genome of *Rhizobium* sp. strain RSm-3 was performed at the Hubbard Center for Genome Studies (University of New Hampshire, Durham, NH) using Illumina techniques (12). A standard Illumina shotgun library was constructed and sequenced using the Illumina HiSeq 2500 platform, which generated 1,585,078 reads (260-bp insert size) totaling 341 Mbp. The Illumina sequence data were trimmed by Trimmonatic version 0.32 (13) and assembled using SPAdes version 3.5 (14), and ALLPaths-LG version r52488 (15). The final draft assembly for *Rhizobium* sp. strain RSm-3 consisted of 60 contigs, with an *N*_50_ contig size of 313.1 kb and 54.3× coverage of the genome. The final assembled genome contained a total sequence length of 6,912,093 bp, with a G+C content of 61.4%.

The assembled RSm-3 genome was annotated via the NCBI Prokaryotic Genome Annotation Pipeline (PGAP) and resulted in 6,511 candidate protein-coding genes, 46 tRNAs, four rRNA (two 5S rRNA, one 16S rRNA, and one 23S rRNA) regions, and 111 (1.69%) pseudogenes. The genome of RSm-3 also revealed the presence of the *nif* and common *nod* operons involved in nitrogen fixation and host plant nodulation, respectively. A total of 590 signal peptide-coding genes and 1,563 enzyme-coding genes were assigned through the annotation program.

There are two major branches of common bean, Mesoamerican and Andean (16), and a third genetic diversification of the common bean is found in the Peru-Ecuador region (17). A new species of *Rhizobium*, *R. ecuadorense*, has been proposed for the microsymbiont of the Peru-Ecuador common bean. The average nucleotide identity (ANI) score for *Rhizobium* sp. strain RSm-3 was 98% similarity with the *R. ecuadorense* type strain (CNPSo 671) (18) suggesting that it is a subspecies of *R. ecuadorense*.

### Accession number(s).

This whole-genome shotgun sequence has been deposited at DDBJ/EMBL/GenBank under the accession number MAWZ00000000. The version described in this paper is the first version, MAWZ01000000.

## References

[1] Moschetti G, Peluso A, Protopapa A, Anastasio M, Pepe O, Defez R 2005 Use of nodulation pattern, stress tolerance, *nodC* gene amplification, RAPD-PCR and RFLP-16S rDNA analysis to discriminate genotypes of *Rhizobium leguminosarum* biovar viciae. Syst Appl Microbiol 28:619–631. doi:10.1016/j.syapm.2005.03.009. 16156120

[2] Mousavi SA, Österman J, Wahlberg N, Nesme X, Lavire C, Vial L, Paulin L, de Lajudie P, Lindström K 2014 Phylogeny of the *Rhizobium*-*Allorhizobium*-*Agrobacterium* clade supports the delineation of *Neorhizobium* gen. nov. Syst Appl Microbiol 37:208–215. doi:10.1016/j.syapm.2013.12.007. 24581678

[3] Long SR 2001 Genes and signals in the *Rhizobium*-legume symbiosis. Plant Physiol 125:69–72. doi:10.1104/pp.125.1.69. 11154299 PMC1539328

[4] Díaz CL, Melchers LS, Hooykaas PJJ, Lugtenberg BJJ, Kijne JW 1989 Root lectin as a determinant of host-plant specificity in the *Rhizobium*-legume symbiosis. Nature 338:579–581. doi:10.1038/338579a0.

[5] Legocki RP, Verma DPS 1980 Identification of nodule-specific host proteins (nodulins) involved in the development of *Rhizobium*-legume symbiosis. Cell 20:153–163. doi:10.1016/0092-8674(80)90243-3. 7388942

[6] Okazaki S, Tittabutr P, Teulet A, Thouin J, Fardoux J, Chaintreuil C, Gully D, Arrighi JF, Furuta N, Miwa H, Yasuda M, Nouwen N, Teaumroong N, Giraud E 2016 *Rhizobium*-legume symbiosis in the absence of *nod* factors: two possible scenarios with or without the T3SS. ISME J 10:64–74. doi:10.1038/ismej.2015.103. 26161635 PMC4681849

[7] Zahran HH 1999 *Rhizobium*-legume symbiosis and nitrogen fixation under severe conditions and in an arid climate. Microbiol Mol Biol Rev 63:968–989. 10585971 10.1128/mmbr.63.4.968-989.1999 PMC98982

[8] Gopalakrishnan S, Sathya A, Vijayabharathi R, Varshney RK, Gowda CLL, Krishnamurthy L 2015 Plant growth promoting rhizobia: challenges and opportunities. 3 Biotech 5:355–377. doi:10.1007/s13205-014-0241-x. PMC4522733 28324544

[9] Christou P 1997 Biotechnology applied to grain legumes. Field Crops Res 53:83–97. doi:10.1016/S0378-4290(97)00024-5.

[10] Junier P, Alfaro M, Guevara R, Witzel KP, Carú M 2014 Genetic diversity of *Rhizobium* present in nodules of *Phaseolus vulgaris* L. cultivated in two soils of the central region in Chile. Appl Soil Ecol 80:60–66. doi:10.1016/j.apsoil.2014.03.014.

[11] Xu BJ, Chang SKC 2008 Total phenolic content and antioxidant properties of eclipse black beans (*Phaseolus vulgaris* L.) as affected by processing methods. J Food Sci 73:H19–H27. doi:10.1111/j.1750-3841.2007.00625.x. 18298732

[12] Bennett S 2004 Solexa Ltd. Pharmacogenomics 5:433–438. doi:10.1517/14622416.5.4.433. 15165179

[13] Bolger AM, Lohse M, Usadel B 2014 Trimmomatic: a flexible trimmer for Illumina sequence data. Bioinformatics 30:2114–2120. doi:10.1093/bioinformatics/btu170. 24695404 PMC4103590

[14] Nurk S, Bankevich A, Antipov D, Gurevich AA, Korobeynikov A, Lapidus A, Prjibelski AD, Pyshkin A, Sirotkin A, Sirotkin Y, Stepanauskas R, Clingenpeel SR, Woyke T, McLean JS, Lasken R, Tesler G, Alekseyev MA, Pevzner PA 2013 Assembling single-cell genomes and mini-metagenomes from chimeric MDA products. J Comput Biol 20:714–737. doi:10.1089/cmb.2013.0084. 24093227 PMC3791033

[15] Gnerre S, MacCallum I, Przybylski D, Ribeiro FJ, Burton JN, Walker BJ, Sharpe T, Hall G, Shea TP, Sykes S, Berlin AM, Aird D, Costello M, Daza R, Williams L, Nicol R, Gnirke A, Nusbaum C, Lander ES, Jaffe DB 2011 High-quality draft assemblies of mammalian genomes from massively parallel sequence data. Proc Natl Acad Sci U S A 108:1513–1518. doi:10.1073/pnas.1017351108. 21187386 PMC3029755

[16] Gepts P 1990 Biochemical-evidence bearing on the domestication of *Phaseolus* (Fabaceae) beans. Econ Bot 44:28–38. doi:10.1007/BF02860473.

[17] Debouck DG, Toro O, Paredes OM, Johnson WC, Gepts P 1993 Genetic diversity and ecological distribution of *Phaseolus vulgaris* (Fabaceae) in northwestern South America. Econ Bot 47:408–423. doi:10.1007/BF02907356.

[18] Ribeiro RA, Delamuta JRM, Gomes DF, Souza RC, Chueire LMO, Hungria M 2015 Genome sequence of Rhizobium ecuadorense strain CNPSo 671T, an indigenous N2-fixing symbiont of the Ecuadorian common bean (Phaseolus vulgaris L.) genetic pool. Genome Announc 3(5):e01058-15. doi:10.1128/genomeA.01058-15. 26383667 PMC4574372

